# Transarterial Embolization for Malignancy-Related Gastrointestinal Bleeding: Outcomes with and Without CT Extravasation

**DOI:** 10.3390/diagnostics16152350

**Published:** 2026-07-27

**Authors:** Kwang Kun Cho, Byung Chan Lee, Hyoung Ook Kim, Chan Park, In Woo Choi, John F. Angle

**Affiliations:** 1 Department of Radiology, Chonnam National University Hwasun Hospital, Hwasun-gun 58128, Republic of Korea; 0509choyoon@gmail.com (K.K.C.);; 2 Department of Radiology, Chonnam National University Medical School, Hwasun-gun 58128, Republic of Korea; 3 Department of Radiology, Chonnam National University Hospital, Gwangju 61469, Republic of Korea; 4 Department of Radiology and Medical Imaging, University of Virginia Health System, 1215 Lee Street, Charlottesville, VA 22908, USA

**Keywords:** gastrointestinal hemorrhage, transarterial embolization, malignancy, contrast extravasation, computed tomography angiography, interventional radiology, shock index

## Abstract

**Background/Objectives**: Gastrointestinal (GI) bleeding in patients with malignancy is frequently refractory to endoscopic and conservative management. Transarterial embolization (TAE) is an established treatment option, but whether the presence of contrast extravasation on preprocedural computed tomography (CT) influences outcomes remains unclear. This study aimed to evaluate the efficacy and safety of TAE for malignancy-related GI bleeding in patients with and without CT-detected contrast extravasation. **Methods**: From January 2012 to December 2023, 40 consecutive patients who underwent TAE for malignancy-related GI bleeding were retrospectively analyzed and categorized into CT extravasation (*n* = 25) and CT no-extravasation (*n* = 15) groups based on preprocedural CT findings. Technical success, clinical success, 30-day mortality, overall survival (OS), and procedure-related complications were compared between groups, and factors associated with OS were evaluated using Cox regression. **Results**: The technical and clinical success rates were 100% (40/40) and 82.5% (33/40), respectively. Clinical success did not differ between the CT extravasation and no-extravasation groups (84.0% [21/25] vs. 80.0% [12/15]; *p* = 0.747). Two of 40 patients (5.0%) died within 30 days, one in each group (*p* = 0.708). OS did not differ significantly (log-rank *p* = 0.707; median, 147 vs. 187 days). A shock index > 1 was independently associated with worse OS (hazard ratio, 2.308; 95% confidence interval, 1.071–4.972; *p* = 0.033). No major procedure-related complications occurred. **Conclusions**: TAE achieved high technical success and comparable short-term clinical outcomes in patients with and without CT extravasation. Hemodynamic instability, rather than CT extravasation, was independently associated with OS.

## 1. Introduction

Malignancy-related gastrointestinal (GI) bleeding is a significant clinical condition that accounts for approximately 2–8% of acute nonvariceal upper GI bleeding and 3–11% of acute lower GI bleeding, as reported in epidemiologic studies [1,2]. It can lead to anemia, hemodynamic instability, and the need for transfusion, often resulting in prolonged hospitalization and adverse clinical outcomes, particularly in patients with advanced cancer [3]. Although endoscopic hemostasis is the first-line treatment for GI bleeding, it may be less effective in tumor-related bleeding because tumor infiltration, friable neovascularity, and extensive mucosal involvement can limit visualization and endoscopic access, potentially resulting in technical failure or recurrent bleeding [4,5]. Furthermore, surgical resection for advanced GI malignancies with active bleeding is associated with substantial perioperative morbidity and mortality, thereby limiting its feasibility in many patients [6,7]. In addition, radiotherapy generally plays a limited role in acute hemodynamically significant bleeding because of its delayed onset [7].

Transarterial embolization (TAE) is a minimally invasive and repeatable option that can provide prompt hemostasis for tumor-related GI bleeding and may serve as a valuable palliative treatment in patients with advanced cancer and limited therapeutic alternatives [6,7,8]. Previous studies have reported high technical success rates and favorable short-term clinical outcomes of TAE for malignancy-related GI bleeding, although the presence of angiographic contrast extravasation varied across cohorts [8,9,10,11,12,13,14,15]. However, because tumor-related hemorrhage is often slow or intermittent, active extravasation may not be visible on diagnostic imaging despite clinical evidence of bleeding [16].

To date, comparative outcome data stratified by preprocedural computed tomography (CT) extravasation status remain limited in the literature [8,9,13]. Published studies have generally reported overall outcomes without stratifying patients according to preprocedural CT findings, and patients without CT-demonstrated extravasation have rarely been analyzed as a distinct subgroup. As a result, the role of embolization in CT-negative patients remains insufficiently defined by directly comparative outcome data. Such outcome comparisons may help inform clinical decision-making and clarify the role of TAE in CT-negative cases. Therefore, this study aimed to compare the technical and clinical success of TAE for malignancy-related GI bleeding between patients with and without preprocedural CT extravasation, with the hypothesis that outcomes would be comparable between groups. The secondary objectives were to compare 30-day mortality, overall survival (OS), and procedure-related complications between groups.

## 2. Materials and Methods

### 2.1. Study Design and Patients

This retrospective, single-center study analyzed data from consecutive patients who underwent TAE for malignancy-related GI bleeding between January 2012 and December 2023. The study was approved by the institutional review board, and the requirement for written informed consent was waived because of the retrospective nature of the study.

Malignancy-related GI bleeding was defined as clinical evidence of hemorrhage (hematemesis, melena, or hematochezia) associated with decreased hemoglobin levels in patients with histopathologically or radiologically confirmed malignancies [11,12]. Eligible patients were those with malignancy (solid tumors or lymphoma) who underwent TAE for malignancy-related GI bleeding and had contrast-enhanced CT within 24 h before TAE. To ensure that the bleeding was directly attributable to the tumor, cases were included only if they met at least one of the following criteria: (1) endoscopic confirmation of tumor invasion and/or tumor-associated ulceration at the suspected bleeding site prior to angiography or (2) CT evidence of an acute intraluminal hematoma and/or active extravasation adjacent to a known malignant mass. Patients were excluded if they met any of the following criteria: (1) bleeding from non-malignant causes (e.g., peptic ulcer disease without tumor involvement or varices); (2) absence of overt clinical signs of GI bleeding; (3) endoscopic hemostasis performed immediately before or after TAE during the same bleeding episode, which could confound the assessment of TAE efficacy; or (4) loss to follow-up.

### 2.2. TAE Procedures

All TAE procedures were performed by three experienced interventional radiologists (each with >5 years of experience in visceral embolization procedures). Before each procedure, preprocedural CT images and endoscopic findings were reviewed, when available, to localize the suspected bleeding focus. All procedures were performed via common femoral artery access under local anesthesia. Diagnostic angiography was performed under fluoroscopic guidance using a standard 5-Fr diagnostic catheter.

In patients with CT-detected active bleeding, angiography and subsequent embolization were directed toward the bleeding-related feeding artery identified on CT. In those without CT-detected active bleeding, angiography was performed to evaluate tumor-feeding arteries based on tumor location on CT. Empiric embolization was defined as embolization performed in the absence of angiographic evidence of active bleeding, targeting the suspected bleeding source based on CT and/or endoscopic localization [17].

Superselective catheterization of the target vessels was attempted whenever feasible using a 2- to 2.4-Fr microcatheter (Parkway, Asahi, Tokyo, Japan; Progreat, Terumo, Tokyo, Japan; or Renegade, Boston Scientific, Marlborough, MA, USA).

The choice of embolic material was determined by the operator based on angiographic findings and material availability. Embolic agents included n-butyl cyanoacrylate (NBCA) (Histoacryl; B. Braun, Melsungen, Germany) mixed with iodized oil (Lipiodol; Guerbet, Aulnay-Sous-Bois, France), microcoils (Concerto; Medtronic, Minneapolis, MN, USA; Interlock; Boston Scientific, Marlborough, MA, USA; Tornado and Nester; Cook Medical, Bloomington, IN, USA); polyvinyl alcohol (PVA) particles (Contour; Boston Scientific, Cork, Ireland); and gelatin sponge particles (EG-gel and IPZA; Engain, Seongnam, South Korea; and Cutanplast; Mascia Brunelli Spa, Milan, Italy). NBCA was mixed with iodized oil at ratios ranging from 1:2 to 1:4, depending on the distance between the microcatheter tip and the target lesion, as well as operator preference. Embolization was continued until complete occlusion of the target vessel and cessation of contrast extravasation in cases with active bleeding or pseudoaneurysm, or until marked reduction in tumor staining in cases without angiographic evidence of active hemorrhage [10,11,12].

### 2.3. Data Collection and Outcome Assessment

Clinical, laboratory, imaging, and procedure-related variables were retrospectively collected from electronic medical records. Baseline patient characteristics included age, sex, body mass index (BMI), comorbidities (hypertension and diabetes mellitus), primary cancer type, and tumor stage. Solid tumors were staged according to the TNM system [18], and the advanced stage for solid tumors was defined as stage IV. Lymphomas were staged according to the Lugano classification system [19]. Preprocedural laboratory variables included hemoglobin level, platelet count, and international normalized ratio (INR). Preprocedural vital signs included systolic blood pressure (SBP) and heart rate. Shock index (SI) was calculated as heart rate divided by SBP, and hemodynamic instability was defined as SI > 1.0, a threshold that has been previously validated for risk stratification in acute GI bleeding [20,21]. Transfusion data were recorded as the number of packed red blood cell (pRBC) units administered within 24 h before and after TAE. Medication use related to bleeding risk, including anticoagulant or antiplatelet therapy, was recorded. The interval between preprocedural contrast-enhanced CT and TAE was recorded.

Preprocedural CT was performed on one of three multidetector CT scanners: Somatom Definition Flash (dual-source, 2 × 64-row; Siemens Healthineers, Forchheim, Germany), Revolution HD (64-row; GE Healthcare, Milwaukee, WI, USA), or LightSpeed VCT (64-row; GE Healthcare, Milwaukee, WI, USA). All examinations were contrast-enhanced CT angiography of the abdomen and pelvis including arterial and portal venous phases; dedicated CT perfusion imaging was not performed. Preprocedural CT images and angiographic findings were independently assessed by two radiologists, and any discrepancies were resolved by consensus. Preprocedural CT assessment focused on the presence or absence of CT-detected active bleeding. CT-detected active bleeding (contrast extravasation) was defined as focal intraluminal contrast pooling that increased in size or attenuation in later phases compared with the arterial phase [22]. Angiographic findings included contrast extravasation, pseudoaneurysm, tumor staining, and vessel lumen irregularities within the tumor-related feeding arteries. Procedure-related variables included the embolized artery and the embolic materials used (NBCA, coils, PVA particles, and gelatin sponge particles).

Technical success was defined as successful embolization with cessation of angiographic contrast extravasation, when present, or marked reduction in tumor staining on completion angiography [23]. Clinical success was defined as resolution of overt GI bleeding within 24 h after TAE, with no recurrent bleeding during the subsequent 30 days [11,23].

Rebleeding was defined as recurrent overt GI bleeding after initial hemostasis requiring additional transfusion or repeat hemostatic intervention [12]. Rebleeding during follow-up was assessed by review of electronic medical records and, when clinically indicated, was further evaluated by endoscopy, contrast-enhanced CT, and/or repeat angiography. Thirty-day mortality was defined as death from any cause within 30 days of the first TAE. OS was defined as the interval between the date of the first TAE and the date of death from any cause. The date of death was confirmed using the National Medical Information System database, and patients without confirmed death were censored at the date last known to be alive. Follow-up duration was calculated from the date of the first TAE to the date of death or, for surviving patients, to the date last known to be alive. Procedure-related complications were recorded and graded according to the Society of Interventional Radiology classification [24].

### 2.4. Statistical Analysis

Continuous variables were assessed for normality using the Shapiro–Wilk test and are presented as means with standard deviations or medians with interquartile ranges, as appropriate, whereas categorical variables are presented as counts with percentages. Comparisons between the CT extravasation and CT no-extravasation groups were performed using the independent-samples *t*-test or Mann–Whitney U test for continuous variables and the chi-square test or Fisher’s exact test for categorical variables, as appropriate. OS was estimated using the Kaplan–Meier method and compared between groups using the log-rank test.

Factors associated with OS were evaluated using Cox proportional hazards regression analysis. Univariable Cox regression was performed for candidate variables. A multivariable Cox model was constructed using the enter method, with CT extravasation status included as the primary variable of interest and with additional adjustment for hemodynamic instability and advanced cancer stage, selected a priori based on clinical relevance. The proportional hazards assumption was assessed using log-minus-log survival plots. Hazard ratios (HRs) are reported with 95% confidence intervals. Statistical significance was defined as a two-sided *p* < 0.05. Analyses were performed using complete cases without imputations. All analyses were performed using IBM SPSS Statistics (version 26.0; IBM Corp., Armonk, NY, USA).

## 3. Results

### 3.1. Patients’ Baseline Characteristics

**Figure 1:**
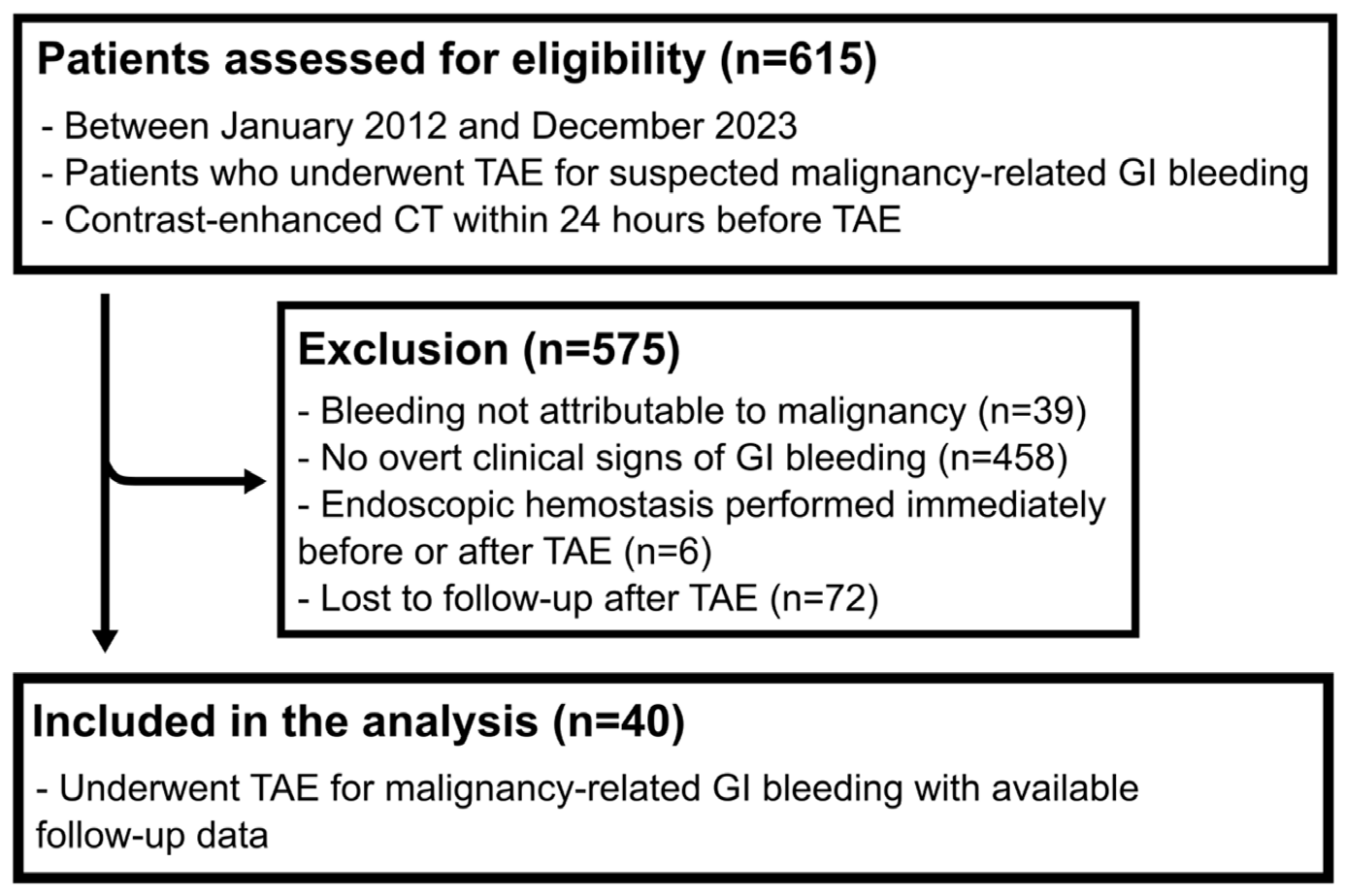
Flow diagram of patient selection.

Forty patients met the inclusion criteria and were included in the analysis, including 25 in the CT extravasation group and 15 in the no-extravasation group (Figure 1). The mean age was 68.9 ± 7.3 years, and 27 patients (67.5%) were male. Comorbidities, including hypertension and diabetes mellitus, were similar between groups. Presenting symptoms included hematemesis in 17 patients (42.5%), hematochezia in 15 (37.5%), and melena in 8 (20.0%). The most common primary tumors were gastric cancer in 12 patients (30.0%) and pancreatic cancer in 11 (27.5%), and 27 patients (67.5%) had stage IV disease. Baseline hemoglobin level was lower in the CT extravasation group than in the CT no-extravasation group (7.1 ± 1.4 vs. 8.2 ± 1.9 g/dL; *p* = 0.038), whereas other baseline characteristics were comparable between groups (Table 1).

### 3.2. Angiographic Findings and Embolic Materials

The most common site of bleeding was the stomach (*n* = 15, 37.5%), followed by the duodenum (*n* = 10, 25.0%), with no significant difference in bleeding location between the CT extravasation and no-extravasation groups. Contrast extravasation and pseudoaneurysm were identified on angiography in 9 of 40 patients (22.5%) and 10 of 40 patients (25.0%), respectively, and were observed exclusively in the CT extravasation group (9/25 vs. 0/15; *p* = 0.015, 10/25 vs. 0/15; *p* = 0.006, respectively). Overall, angiographic signs of active bleeding (contrast extravasation or pseudoaneurysm) were present in 18 of the 25 patients (72.0%) in the CT extravasation group. Tumor staining was more frequent in the CT no-extravasation group than in the CT extravasation group (12/15 [80.0%] vs. 12/25 [48.0%]; *p* = 0.094). Vessel lumen irregularity was observed in 10 patients (25.0%) and did not differ significantly between the groups (5/25 [20.0%] vs. 5/15 [33.3%]; *p* = 0.457). In the CT no-extravasation group, tumor staining and/or vessel lumen irregularities were observed in all patients (15/15, 100%). Embolic materials, including PVA particles, gelatin sponge particles, coils, and NBCA, were used, with no significant between-group differences (Table 2).

### 3.3. Technical and Clinical Outcomes

Technical success was achieved in all patients (100%). Clinical success was achieved in 33 of 40 patients (82.5%), including 21 of 25 (84.0%) in the CT extravasation group and 12 of 15 (80.0%) in the CT no-extravasation group (*p* = 0.747) (Table 3). Thirty-day mortality was 2 of 40 patients (5.0%), with one death in each group (*p* = 0.708) (Table 3). Both patients who died within 30 days died from progression of the underlying malignancy, without documented recurrent GI bleeding, and neither death was related to the embolization procedure. Post-TAE laboratory parameters and hemodynamic indices, including SI, did not significantly differ between the groups (Table 3). During follow-up, rebleeding occurred in 7 of 40 patients (17.5%), with no significant between-group differences (Table 3). Among patients with rebleeding, the median time to first rebleeding was 33 days (IQR, 7–128 days). The interval between preprocedural CT and TAE did not differ significantly between patients with and without clinical success (3.8 h [IQR, 1.7–9.2] vs. 2.1 h [IQR, 1.8–2.8]; *p* = 0.165). No procedure-related complications were identified.

### 3.4. OS and Cox Regression

**Figure 2:**
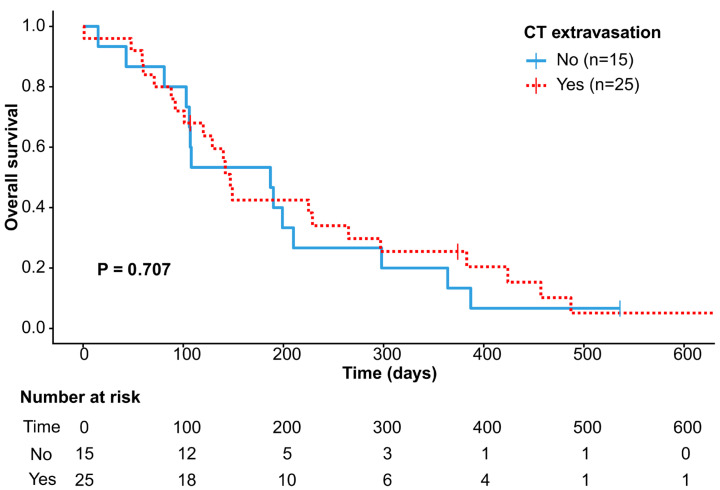
Kaplan–Meier curves for overall survival stratified by CT extravasation status. Survival was compared using the log-rank test (*p* = 0.707). Numbers at risk are shown below the plot; tick marks indicate censored observations.

The median follow-up duration was 144 days (IQR, 99–297; range, 1–699). Three patients remained alive and were censored at 107, 374, and 536 days after TAE. During the entire follow-up period, 37 of 40 patients (92.5%) died, including 23 of 25 (92.0%) in the CT extravasation group and 14 of 15 (93.3%) in the CT no-extravasation group. OS did not differ significantly between the CT extravasation and CT no-extravasation groups (log-rank *p* = 0.707) (Figure 2). The Kaplan–Meier-estimated median OS was 147 days (95% CI, 132.94–161.06) in the CT extravasation group and 187 days (95% CI, 82.22–291.78) in the CT no-extravasation group. The median OS for the entire cohort was 147 days (95% CI, 77.22–216.78). In the multivariable Cox regression analysis, an SI > 1 was independently associated with worse OS (HR, 2.308; 95% CI, 1.071–4.972; *p* = 0.033), whereas CT-detected active bleeding and stage IV disease did not reach statistical significance (Table 4).

## 4. Discussion

This study found that the outcomes after TAE for malignancy-related GI bleeding were comparable between patients with and without CT-detected extravasation. Technical and clinical success and OS did not differ according to CT extravasation status. Thirty-day mortality was low and did not differ between the groups. Multivariable analysis identified hemodynamic instability as an independent predictor of worse OS, whereas CT-detected extravasation was not. Stage IV disease was not significantly associated with OS, which may reflect the limited sample size and heterogeneity of this predominantly advanced-stage cohort. Although several retrospective studies have reported outcomes after TAE for malignancy-related GI bleeding, comparative outcome data stratified by preprocedural CT extravasation status remain limited [8,9,13]. A clinically relevant aspect of this study is the explicit stratification of patients by the presence or absence of preprocedural CT extravasation, with outcomes reported for each subgroup; the cohort predominantly comprised patients with advanced-stage malignancy, reflecting a real-world population with often limited therapeutic options.

Endoscopic hemostasis remains the first-line and most effective approach in most cases of GI bleeding [25]. However, in advanced malignancies, tumor infiltration and friable neovascularity can limit visualization and endoscopic access, making durable hemostasis challenging and predisposing patients to recurrent bleeding [4,6]. Surgical and radiotherapeutic approaches are frequently impractical or delayed in acute settings, particularly in patients with limited physiological reserves and palliative care goals [6,26]. In this context, TAE offers a minimally invasive and repeatable option for prompt hemorrhage control and may be considered when endoscopic therapy is unsuccessful or not feasible [8,15]. These findings support its role by demonstrating comparable short-term outcomes in patients with and without CT-detected extravasations.

A prior series of TAE for malignancy-related GI bleeding reported technical success rates of approximately 85–100% and short-term clinical success rates of approximately 52–78%, although outcome definitions and follow-up windows varied across studies [8,9,10,15,27]. In this cohort, technical success was achieved in all patients, and the 30-day clinical success rate was 82.5%, which was numerically higher than rates reported in several previous studies; however, direct cross-study comparisons are limited by heterogeneity in definitions, patient selection, and tumor spectrum. Reported clinical success may also have varied because the cohorts included different tumor types and bleeding phenotypes, and procedural factors may also have contributed. The high technical success observed in this cohort may reflect careful patient selection, as all included cases had a clinically and/or radiologically identifiable target territory, as well as the use of superselective embolization whenever feasible. No procedure-related complications were identified in the analyzed cohort, consistent with the low complication rates reported in previous retrospective series [8,9].

When angiography does not reveal definite signs of active bleeding, empirical embolization targeting suspected tumor-feeding arteries may be performed based on CT and/or endoscopic localization, with angiographic surrogates such as tumor staining or vessel lumen irregularity used to guide target selection in CT-negative presentations [11,15]. Tumor-related GI bleeding is often diffuse and intermittent; therefore, discrete contrast extravasation may be absent on CT/CT angiography despite clinically significant bleeding [4,16,28]. CTA may also have limited sensitivity for slow or low-volume bleeding [29,30,31]. Because tumor-related bleeding arises from fragile neovessels that may give rise to pseudoaneurysms and are unlikely to undergo effective vasoconstrictive tamponade, spontaneous durable hemostasis is unlikely in the absence of targeted therapy [4,6,15,16].

In our cohort, positive CT findings were not always accompanied by definite angiographic evidence of active bleeding. In this study, embolization was performed in all cases in which the tumor was considered the likely source of bleeding. This strategy can reduce arterial inflow to the bleeding tumor bed and diminish tumor staining. Previous studies have reported clinically meaningful short-term hemostasis even when a discrete bleeding focus was not angiographically demonstrated [8,11]. However, the effectiveness of this approach may vary, and non-target ischemia remains a consideration, particularly with less selective embolization and/or the use of deeply penetrating embolic agents [32,33]. Beyond the agents used in this cohort, the ongoing evolution of embolic materials, including non-adhesive liquid embolic agents, has further expanded endovascular treatment options for complex visceral and tumor-related hemorrhage, allowing embolization strategies to be tailored according to vascular anatomy and bleeding characteristics [34]. In this cohort, short-term outcomes were similar between the CT-positive and CT-negative groups, suggesting that reduction in arterial inflow to the tumor bed may achieve hemostasis even in the absence of a discrete bleeding point.

This study has several limitations. First, its retrospective, single-center design and relatively small sample size may limit generalizability and statistical power. The multivariable results should therefore be interpreted cautiously. Second, 72 patients who underwent TAE during the study period were excluded because adequate follow-up data for outcome ascertainment were unavailable, a number nearly twice that of the analyzed cohort. This may have introduced substantial attrition bias, and the reported technical success, clinical success, and complication rates should therefore be interpreted as applying to patients with documented follow-up rather than to all patients undergoing TAE for malignancy-related GI bleeding. Third, embolic materials and procedural techniques were not standardized and reflected operator preference, and tumor types and primary bleeding sites were heterogeneous. Fourth, variability in CT protocols, contrast phases, and image acquisition timing may have affected the detection of contrast extravasation. Because the time of bleeding onset could not be reliably determined in this retrospective cohort, the interval between preprocedural CT and TAE does not reflect the total delay from hemorrhage onset to intervention. Fifth, the absence of a non-TAE control group precludes causal inferences regarding the effect of TAE on outcomes, particularly in patients without CT extravasation. Although spontaneous durable hemostasis is unlikely in tumor-related bleeding, the efficacy of TAE in CT-negative cases remains uncertain in this cohort because the contribution of concurrent supportive care, including transfusion and correction of coagulopathy, to early stabilization could not be quantified. Finally, follow-up was limited, and the small number of rebleeding events restricted the assessment of predictors of clinical failure. Larger studies with appropriate control groups are needed to better define the safety and efficacy of empiric or prophylactic embolization in this setting.

In conclusion, TAE showed comparable technical success, clinical success, 30-day mortality, and OS in patients with malignancy-related GI bleeding with and without preprocedural CT extravasation. Hemodynamic instability was independently associated with poorer outcomes. Although this single-arm study does not allow conclusions regarding superiority over conservative management, the findings suggest that TAE may represent a reasonable therapeutic option for patients with clinically suspected malignancy-related GI bleeding and limited therapeutic alternatives, even when CT does not demonstrate extravasation.

## Figures and Tables

**Table 1:** Patients’ baseline characteristics.

Characteristics	Total (*n* = 40)	CT Extravasation (*n* = 25)	CT No-Extravasation (*n* = 15)	*p*-Value
Age (years)	68.9 ± 7.3	68.7 ± 7.6	69.3 ± 7.1	0.730
Male sex	27 (67.5)	16 (64.0)	11 (73.3)	0.340
Body mass index (kg/m^2^)	21.4 ± 3.9	20.9 ± 3.9	22.1 ± 4.1	0.373
Hypertension	10 (25.0)	7 (28.0)	3 (20.0)	0.715
Diabetes mellitus	10 (25.0)	7 (28.0)	3 (20.0)	0.715
Presenting symptoms				
Hematemesis	17 (42.5)	10 (40.0)	7 (46.7)	0.680
Hematochezia	15 (37.5)	9 (36.0)	6 (40.0)	0.800
Melena	8 (20.0)	6 (24.0)	2 (13.3)	0.686
Prior GI bleeding	4 (10.0)	2 (8.0)	2 (13.3)	0.622
Anticoagulant or antiplatelet use	2 (5.0)	0 (0.0)	2 (13.3)	0.135
Primary tumor type				0.975
Gallbladder cancer	1 (2.5)	0 (0.0)	1 (6.7)	
Pancreatic cancer	11 (27.5)	8 (32.0)	3 (20.0)	
Duodenal cancer	2 (5.0)	1 (4.0)	1 (6.7)	
Rectal cancer	4 (10.0)	2 (8.0)	2 (13.3)	
Cervical cancer	1 (2.5)	1 (4.0)	0 (0.0)	
Gastric cancer	12 (30.0)	7 (28.0)	5 (33.3)	
Lymphoma	2 (5.0)	1 (4.0)	1 (6.7)	
Hepatocellular carcinoma	3 (7.5)	2 (8.0)	1 (6.7)	
Esophageal cancer	3 (7.5)	2 (8.0)	1 (6.7)	
Cholangiocarcinoma	1 (2.5)	1 (4.0)	0 (0.0)	
Tumor stage				0.522
Stage I or II	4 (10.0)	3 (12.0)	1 (6.7)	
Stage III	9 (22.5)	7 (28.0)	2 (13.3)	
Stage IV	27 (67.5)	15 (60.0)	12 (80.0)	
Baseline laboratory variables				
Hemoglobin (g/dL)	7.5 ± 1.7	7.1 ± 1.4	8.2 ± 1.9	0.038
Platelet count (×10^3^/μL)	191.5 ± 79.5	182.9 ± 77.3	205.6 ± 83.6	0.390
INR	1.24 (1.09–1.56)	1.37 (1.12–1.67)	1.17 (1.07–1.29)	0.111
pRBC transfusion (units)	3 (1–5)	3 (1.5–5.5)	2 (1–4)	0.422
SBP < 90 mm Hg	14 (35.0)	11 (44.0)	3 (20.0)	0.123
Heart rate > 100 bpm	17 (42.5)	11 (44.0)	6 (40.0)	0.804
SI > 1.0	21 (52.5)	15 (60.0)	6 (40.0)	0.220
pRBC transfusion > 10 units	8 (20.0)	5 (20.0)	3 (20.0)	1.000
CT to TAE interval (h)	3.5 (1.7–7.3)	3.1 (1.6–5.7)	4.0 (2.1–7.5)	0.567

Data are presented as mean ± standard deviation, median (interquartile range), or number (%). Abbreviations: CT, computed tomography; h, hours; INR, international normalized ratio; pRBC, packed red blood cells; SBP, systolic blood pressure; SI, shock index; TAE, transarterial embolization.

**Table 2:** Angiographic findings and embolic materials.

Variables	Total (*n* = 40)	CT Extravasation (*n* = 25)	CT No-Extravasation (*n* = 15)	*p*-Value
Bleeding location				0.505
Stomach	15 (37.5)	9 (36.0)	6 (40.0)	
Duodenum	10 (25.0)	6 (24.0)	4 (26.7)	
Jejunum	3 (7.5)	3 (12.0)	0 (0.0)	
Ileum	2 (5.0)	0 (0.0)	2 (13.3)	
Colon/rectum	5 (12.5)	3 (12.0)	2 (13.3)	
Esophagus	3 (7.5)	2 (8.0)	1 (6.7)	
Bile duct	2 (5.0)	2 (8.0)	0 (0.0)	
Angiographic findings				
Extravasation	9 (22.5)	9 (36.0)	0 (0.0)	0.015
Pseudoaneurysm	10 (25.0)	10 (40.0)	0 (0.0)	0.006
Tumor staining	24 (60.0)	12 (48.0)	12 (80.0)	0.094
Vessel lumen irregularity	10 (25.0)	5 (20.0)	5 (33.3)	0.457
Embolic materials				
PVA particles	4 (10.0)	3 (12.0)	1 (6.7)	1.000
Gelatin sponge particles	12 (30.0)	6 (24.0)	6 (40.0)	0.311
Coils + particles	10 (25.0)	8 (32.0)	2 (13.3)	0.269
Coils	16 (40.0)	10 (40.0)	6 (40.0)	1.000
NBCA	4 (10.0)	3 (12.0)	1 (6.7)	1.000

Data are presented as number (%). Abbreviations: NBCA, n-butyl cyanoacrylate; PVA, polyvinyl alcohol.

**Table 3:** Post-TAE outcomes and laboratory parameters.

Variables	Total (*n* = 40)	CT Extravasation (*n* = 25)	CT No-Extravasation (*n* = 15)	*p*-Value
Technical success	40 (100)	25 (100)	15 (100)	
Clinical success	33 (82.5)	21 (84.0)	12 (80.0)	0.747
30-day mortality	2 (5.0)	1 (4.0)	1 (6.7)	0.708
Rebleeding during follow-up	7 (17.5)	3 (12.0)	4 (26.7)	0.392
Post-TAE laboratory variables				
Hemoglobin (g/dL)	9.5 ± 1.4	9.4 ± 1.6	9.7 ± 0.9	0.498
Platelet count (×10^3^/μL)	216.4 ± 91.8	200.6 ± 97.6	242.7 ± 77.3	0.163
INR	1.15 (1.04–1.30)	1.20 (1.04–1.34)	1.10 (1.05–1.29)	0.665
Shock index	0.68 ± 0.19	0.68 ± 0.22	0.67 ± 0.12	0.919
SBP (mm Hg)	123.6 ± 13.5	125.6 ± 13.8	120.1 ± 12.7	0.216
Heart rate (bpm)	78.5 (72–88)	80 (72–87.5)	78 (70–88)	0.484

Data are presented as mean ± standard deviation, median (interquartile range), or number (%). Abbreviations: INR, international normalized ratio; SBP, systolic blood pressure; TAE, transarterial embolization.

**Table 4:** Cox proportional hazards model for overall survival.

		Univariable Analysis		Multivariable Analysis	
Category		Hazard Ratio	*p*-Value	Hazard Ratio	*p*-Value
Age		1.011 (0.955, 1.071)	0.700		
Sex	Female	Reference			
	Male	0.762 (0.368, 1.575)	0.463		
Anticoagulant	No	Reference			
	Yes	0.539 (0.187, 1.553)	0.252		
CT-detected active bleeding	No	Reference			
	Yes	0.879 (0.448, 1.724)	0.708	0.639 (0.303, 1.348)	0.240
Hemoglobin		0.872 (0.701, 1.084)	0.218		
Hemodynamic instability	SI ≤ 1.0	Reference			
	SI > 1.0	1.960 (0.996, 3.858)	0.051	2.308 (1.071, 4.972)	0.033
Advanced stage	Stage I–III	Reference			
	Stage IV	1.256 (0.610, 2.583)	0.536	1.051 (0.499, 2.216)	0.896
pRBC transfusion	≤10 units	Reference			
	>10 units	1.020 (0.442, 2.354)	0.964		

Hazard ratios are presented with 95% confidence intervals in parentheses. Abbreviations: CT, computed tomography; SI, shock index.

## Data Availability

The original contributions presented in this study are included in the article. Further inquiries can be directed to the corresponding author.

## Abbreviations

The following abbreviations are used in this manuscript:

BMI	Body mass index
CI	Confidence interval
CT	Computed tomography
GI	Gastrointestinal
HR	Hazard ratio
INR	International normalized ratio
IQR	Interquartile range
NBCA	N-butyl cyanoacrylate
OS	Overall survival
PVA	Polyvinyl alcohol
SBP	Systolic blood pressure
SI	Shock index
TAE	Transarterial embolization
TNM	Tumor–node–metastasis
